# Developing a Virtual Reality Simulation System for Preoperative Planning of Robotic-Assisted Thoracic Surgery

**DOI:** 10.3390/jcm13020611

**Published:** 2024-01-21

**Authors:** Hideki Ujiie, Ryohei Chiba, Aogu Yamaguchi, Shunsuke Nomura, Haruhiko Shiiya, Aki Fujiwara-Kuroda, Kichizo Kaga, Chad Eitel, Tod R. Clapp, Tatsuya Kato

**Affiliations:** 1 Department of Thoracic Surgery, Hokkaido University Hospital, Sapporo 060-8648, Hokkaido, Japan; chiba.ryohei.x2@elms.hokudai.ac.jp (R.C.); owl_rampage@yahoo.co.jp (S.N.); hshiiya@outlook.jp (H.S.); a-black@wj8.so-net.ne.jp (A.F.-K.); kagahmg@yahoo.co.jp (K.K.); katotatu7@msn.com (T.K.); 2 Division of Radiology, Department of Medical Technology, Hokkaido University Hospital, Sapporo 060-8648, Hokkaido, Japan; aogu@huhp.hokudai.ac.jp; 3 Department of Biomedical Sciences, College of Veterinary Medicine and Biomedical Sciences, Colorado State University, Fort Collins, CO 80523, USA; chad.eitel@colostate.edu (C.E.); tod.clapp@colostate.edu (T.R.C.)

**Keywords:** virtual reality (VR), robotic-assisted thoracic surgery (RATS), head-mounted display (HMD), segmentectomy, three-dimensional reconstruction, image guided surgery

## Abstract

**Background**. Robotic-assisted thoracic surgery (RATS) is now standard for lung cancer treatment, offering advantages over traditional methods. However, RATS’s minimally invasive approach poses challenges like limited visibility and tactile feedback, affecting surgeons’ navigation through com-plex anatomy. To enhance preoperative familiarization with patient-specific anatomy, we devel-oped a virtual reality (VR) surgical navigation system. Using head-mounted displays (HMDs), this system provides a comprehensive, interactive view of the patient’s anatomy pre-surgery, aiming to improve preoperative simulation and intraoperative navigation. **Methods**. We integrated 3D data from preoperative CT scans into Perspectus VR Education software, displayed via HMDs for in-teractive 3D reconstruction of pulmonary structures. This detailed visualization aids in tailored preoperative resection simulations. During RATS, surgeons access these 3D images through Tile-Pro^TM^ multi-display for real-time guidance. **Results**. The VR system enabled precise visualization of pulmonary structures and lesion relations, enhancing surgical safety and accuracy. The HMDs offered true 3D interaction with patient data, facilitating surgical planning. **Conclusions**. VR sim-ulation with HMDs, akin to a robotic 3D viewer, offers a novel approach to developing robotic surgical skills. Integrated with routine imaging, it improves preoperative planning, safety, and accuracy of anatomical resections. This technology particularly aids in lesion identification in RATS, optimizing surgical outcomes.

## 1. Introduction

Surgery remains the cornerstone treatment for primary malignant lung lesions. While conventional thoracotomy has long been the standard approach, recent years have seen a shift towards minimally invasive surgeries, such as video-assisted thoracoscopic surgery (VATS) and robotic-assisted thoracoscopic surgery (RATS). This transition is largely attributed to continual advancements in surgical techniques and technologies. VATS and RATS are now being employed for increasingly complex procedures such as anatomical segmentectomy. However, these minimally invasive methods, particularly RATS, come with their own set of challenges. The lack of stereoscopic vision and reduced tactile feedback in RATS can potentially lead to intraoperative or postoperative complications, such as pneumonia, bleeding, and decreased pulmonary function [1]. Accordingly, the accurate sketch of pulmonary anatomy is of paramount importance for the success of RATS, underscoring the need for precise and detailed preoperative planning.

Preoperative surgical planning utilizing interactive three-dimensional (3D) computed tomography (CT) reconstruction has proven to be a valuable tool in enhancing a surgeon’s understanding of patient-specific anatomy. This method is particularly effective in identifying anatomic variants, which is crucial for precise surgical planning. Furthermore, the feasibility of intraoperative 3D navigation has been demonstrated, offering significant contributions to the safety and accuracy of anatomical resections. These advancements in surgical technology and technique, as evidenced in studies [2,3,4], underscore the continuous evolution towards more precise and patient-tailored thoracic surgeries.

Imaging software programs capable of reconstructing two-dimensional (2D) CT data into 3D visualizations have demonstrated significant advantages in surgical planning, particularly for the pulmonary vessels and bronchi [3,5]. These 3D visualizations aid surgeons in obtaining a more comprehensive understanding of the patient’s anatomy. However, a notable limitation of 3D CT is its static nature; it does not yet offer dynamic simulations that can mimic the intraoperative deformation of the lung. Addressing this gap, several recent reports have highlighted the innovative application of virtual reality (VR) simulation as a surgical training tool [6,7]. VR technology offers a more dynamic and interactive experience, potentially revolutionizing surgical preparation and training by providing simulations that more closely resemble real-life surgical scenarios.

VR navigation utilizing head-mounted displays (HMDs) offers a profoundly immersive experience wherein the user’s entire field of vision is enveloped by a digital image. This immersive environment is further enhanced by displaying slightly different images to each eye, creating a stereoscopic effect that adds depth to the visual experience. The use of advanced volume rendering techniques plays a crucial role in this process, enabling the transformation of medical images into more realistic and detailed representations. This not only improves the visual quality but also turns these images into a powerful communication tool, facilitating a deeper understanding of complex medical data.

We have developed an innovative tool for intraoperative surgical navigation, utilizing the Perspectus VR Education software (https://perspectustech.com/), a networked, multiuser VR platform designed for HMDs [4]. This advanced software enables users to visualize human anatomical models and medical images more effectively, greatly enhancing the creation of instructional content and clinical evaluations. One of the key features of this platform is its networking capabilities, which allow multiple clinicians to collaborate and interact in real time within a shared virtual space. This collaborative environment is particularly beneficial for engaging with other specialists and offers a more meaningful way to interact with medical and scientific l imaging data. Beyond the educational advantages, the use of 3D VR models in clinical settings has shown tangible benefits. According to recent findings [8], these models have contributed to reductions in estimated blood loss, operative time, and length of hospital stay, underscoring the significant impact of VR technology in improving surgical outcomes.

While previous research has validated the benefits of 3D imaging in enhancing understanding and surgical planning for VATS [4], its efficacy in RATS remains less clear. Addressing this gap, our study aimed to develop and evaluate a novel VR system with HMDs. This system is designed to generate virtual dynamic images by volumizing patient-specific CT data, thereby facilitating more effective surgical planning for RATS. Additionally, we aimed to assess the utility of using specific 3D reconstruction images within the surgeon’s console, employing the TilePro™ multi-display input, to enhance the execution of surgical procedures. Our goal is to determine whether this innovative VR simulation system can improve the planning and performance of RATS, potentially leading to better surgical outcomes.

In this study, we would like to develop and evaluate a novel VR system specifically for RATS, and the use of specific 3D reconstruction images in the surgeon’s console is a clear demonstration of the study’s focus on improving surgical techniques and outcomes.

## 2. Materials and Methods

This study was meticulously conducted in strict accordance with the ethical standards set forth in the Declaration of Helsinki, ensuring the highest level of ethical compliance. The research protocol was thoroughly reviewed and approved by the Research Ethics Board of Hokkaido University Hospital, Japan (018-0396), categorizing it as a single-center, phase I feasibility trial. A crucial aspect of our ethical adherence was obtaining informed consent from all patients involved. This consent was specifically for the use of the Perspectus VR Education software to reconstruct their medical data, as detailed in our previous publication [4]. This process ensured that all participants were fully informed about the nature of the study and the use of their data, upholding the principles of ethical research.

Patients scheduled for this study underwent a preoperative contrast-enhanced CT scan. This procedure involved the intravenous administration of an iodinated contrast medium, dosed at 300 mg of iodine per kilogram of body weight, using a mechanical injector (Dual Shot GX; Nemoto Kyorindo, Tokyo, Japan). The contrast medium was injected into an upper extremity vein over a period of 15 s, followed by a 30 mL saline flush to ensure optimal distribution. The CT scans were then performed using an area detector CT system (ADCT; Aquilion ONE; Canon, Tokyo, Japan). The resulting images were saved in the Digital Imaging and Communications in Medicine (DICOM) format on a server client-type workstation (Zio Station 2; AMIN, Tokyo, Japan). From this workstation, the 3D imaging data, including the DICOM files, were extracted. We separately saved the DICOM data for the lung, the pulmonary vessels (specifically the pulmonary artery [PA] and pulmonary vein [PV]), bronchi, and tumors to facilitate detailed analysis and reconstruction.

The DICOM datasets obtained from the CT scans were imported into Fiji, an open-source image processing package (available at https://fiji.sc/ accessed on 1 September 2021.), which is widely used in the scientific community for such analyses. In Fiji, we utilized automated centerline detection algorithms to accurately identify and merge the data of the PA, PV, and bronchi. This process allowed for a detailed and precise reconstruction of these structures. Following this, the processed data was then imported into the Perspectus VR Education software. This import was facilitated by a dedicated macro plugin specifically designed to integrate the data seamlessly into the VR environment. The Perspectus software then enabled us to utilize this data for advanced VR simulations, enhancing our ability to visualize and interact with the patient-specific anatomical structures.

The Perspectus VR Education software platform was performed as a high-performance Hewlett Packard Elite Desk workstation, specifically chosen for its robust capabilities. This workstation was supplied with an Intel i7-8700 CPU (Intel Corporation, Santa Clara CA, USA), 32GB of RAM and an NVIDIA RTX2080 GPU (NVIDIA Corporation, Santa Clara, CA, USA) and ran on Windows 10 Pro, ensuring smooth and efficient processing of complex VR simulations. For the immersive VR experience, we utilized the Samsung Odyssey+ as the HMD. This HMD was integral in providing a high-quality, immersive visual experience necessary for detailed anatomical examination. The Perspectus VR Education software platform is designed to render patient-specific virtual models, which it does by importing and processing the CT data output. This functionality allows for the creation of highly accurate and detailed 3D representations of patient anatomy, essential for precise surgical planning and training.

**Figure 1:**
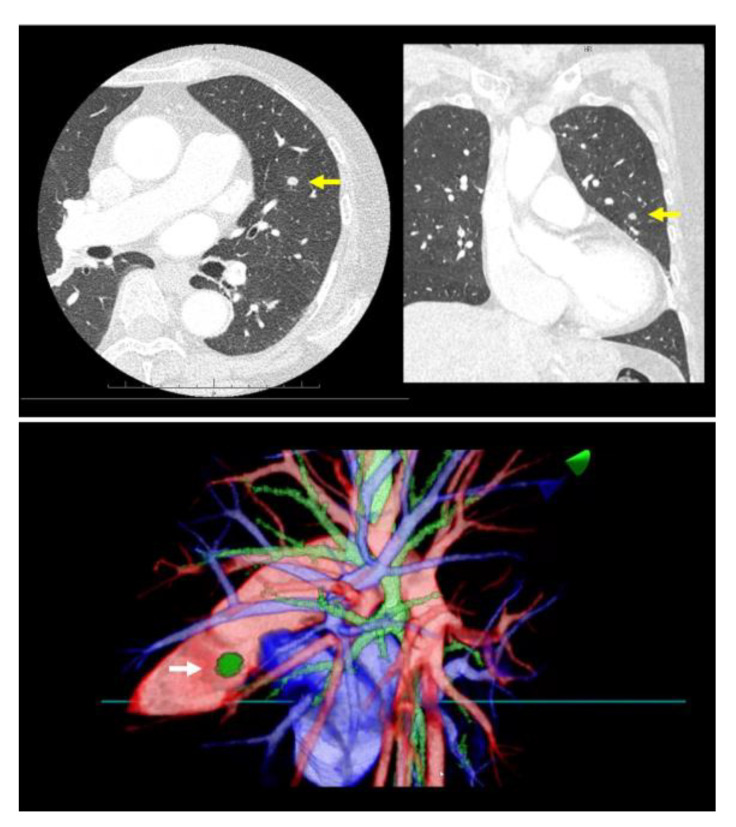
A virtual dynamic image is meticulously generated from patient-specific three-dimensional computed tomography (3D-CT) data, providing a detailed and interactive representation of the patient’s internal anatomy. The top row of images showcases a preoperative chest CT scan of a patient with a left lingular tumor (yellow arrow). The bottom row of images presents representative views of the 3D model with a left lingular tumor (white arrow) derived from the same CT data.

Prior to surgery, a thorough analysis of the surgical resection plan was conducted using patient-specific 3D images. For instance, in a case involving a left lingular tumor, the preoperative chest CT scan and subsequent 3D modeling clearly delineated the tumor’s spatial relationship with surrounding pulmonary structures (as shown in Figure 1). During this review process, the surgeon utilized a HMD to immerse themselves in the virtual reality representation of the patient’s anatomy. The use of hand device controllers enabled the surgeon to interact with the virtual objects in various ways, such as translating, rotating, scaling, cropping, or erasing parts of the model for a detailed examination.

**Figure 2:**
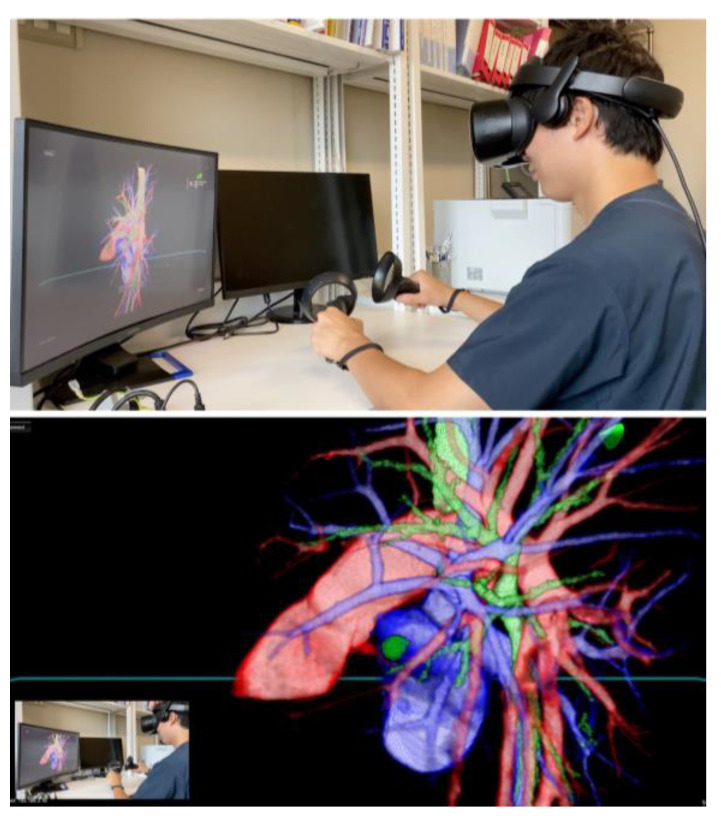
In the preoperative simulation of a left lingular segmentectomy, the surgeon is equipped with a head-mounted display (HMD) to immerse themselves in a virtual reality (VR) environment. This setup allows for the visualization of the surgical site in true three dimensions, offering a comprehensive and detailed view of the patient’s anatomy.

**Figure 3:**
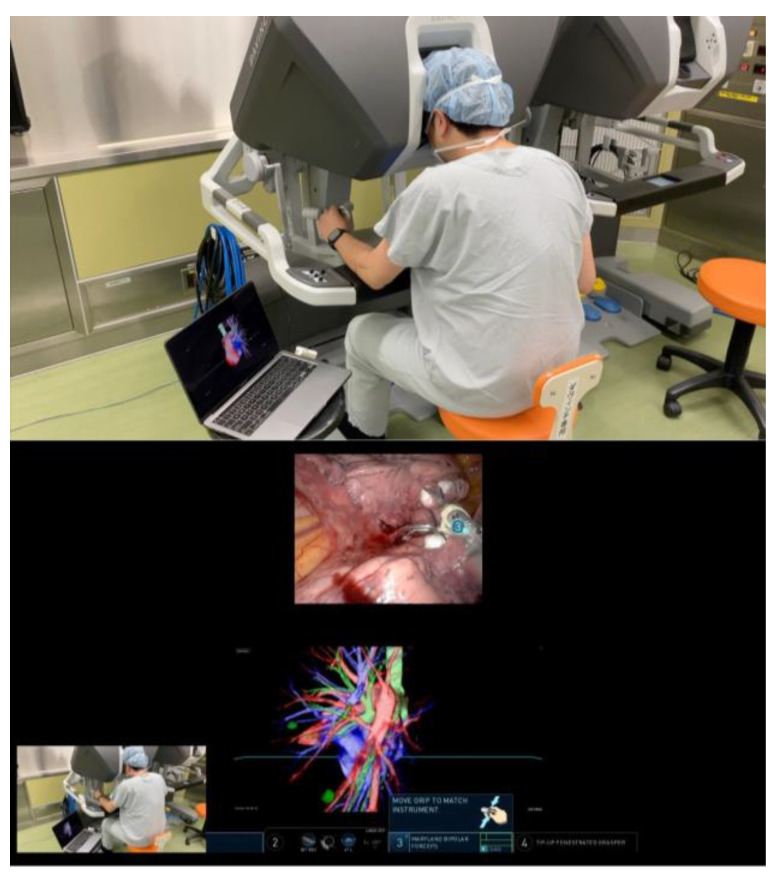
Intraoperative viewing of the 3D VR models was performed at the surgeon’s discretion, allowing for a tailored approach to each surgical procedure.

In preparation for RATS segmentectomy, demanding segmental vessels and bronchi linked with the target segment were identified and examined in detail (illustrated in Figure 2 and Video S1). A specialized system was employed to overlay these virtual data onto the endoscopic video feed displayed by the remote da Vinci surgical console (Intuitive, Sunnyvale, CA, USA). This integration was achieved using TilePro™ (BK Robotic Drop-In US Transducer 8826) (Intuitive Surgical, Inc. Sunnyvale, CA, USA). The intraoperative viewing of these 3D VR models was performed at the surgeon’s foresight to enhance the surgical view and aid in decision-making (as depicted in Figure 3 and Video S2).

## 3. Results

The surgical operation was conducted with the patient placed in the lateral decubitus position, under general anesthesia, with one-lung ventilation. To facilitate the RATS, four da Vinci ports (two 8 mm and two 12 mm) were strategically placed in the 8th intercostal space. Additionally, a 3 cm utility incision, made without rib spreading in the 5th intercostal space, accommodated a 12 mm AirSeal^®^ port (ConMed, Utica, NY, USA) used as an assist port. The entire operation was performed using RATS.

**Figure 4:**
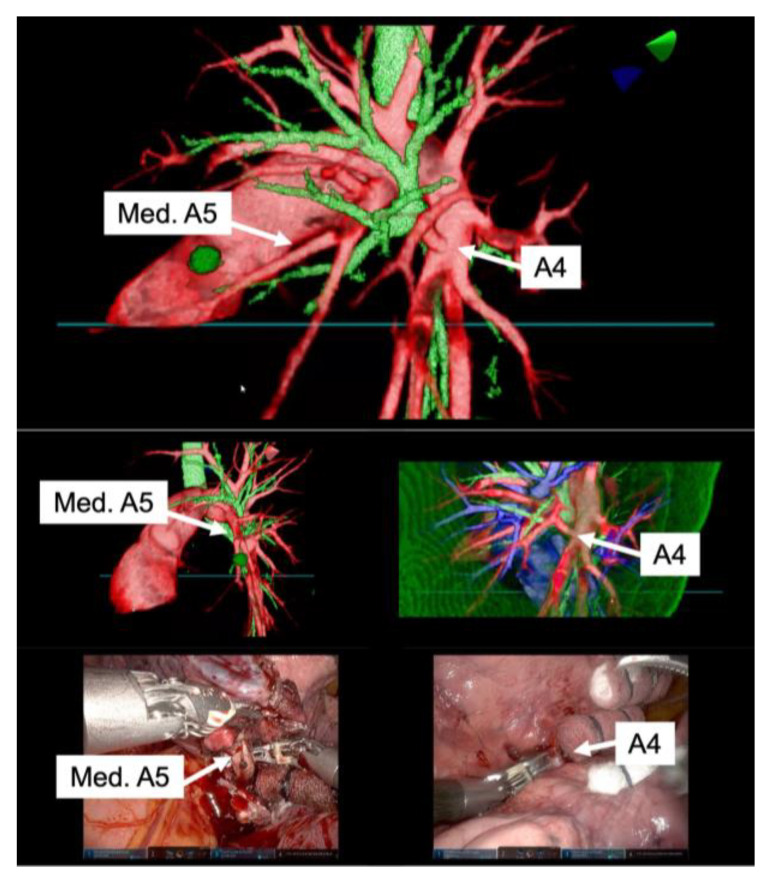
Correlation between preoperative reconstruction and intraoperative findings. The top row of representative images from the 3D model showcases high-resolution anatomical details, including the variant branches such as the mediastinal branch A5 (Med. A5) and the interlobar branch A4. The bottom row of images presents intraoperative views, confirming that the orientation and structure of the pulmonary artery branches, as visualized in the 3D model, were accurately represented.

High-resolution anatomical details of the subsegmental pulmonary artery branches were clearly visualized in the representative images of the 3D model. This detailed visualization was crucial in confirming the orientation of the vessels intraoperatively, both before and after vessel ligation, as depicted in Figure 3. The specific case of left lingular segmentectomy involved the resection of a 15 mm nodular lesion in the left segment 4 + 5. The preoperative preparation with the VR HMD was instrumental in identifying a variant in the PA vessel anatomy, specifically a mediastinal branch A5 (Med. A5) (Figure 4).

During the operation, we employed the Firefly™ fluorescence imaging system (Intuitive, Sunnyvale, CA, USA) for indocyanine green (ICG) fluorescence. This technique facilitated the demarcation of the intersegmental border and the resected segment, following the ligation of relevant PA branches and the injection of ICG (0.25 mg/kg). This integration of imaging technology was key in accurately localizing the intersegmental plane. Subsequently, the lung parenchyma was divided along the marked intersegmental border using endoscopic staples, a process detailed in Video S3.

## 4. Discussion

This study represents the first report of preoperative planning for anatomic lung segmentectomy using a HMD-based VR system in RATS. Our findings, based on the generation of high-resolution, patient-specific 3D lung models using the Perspectus VR Education software, demonstrate significant advancements in surgical planning and execution. These models, which can be freely manipulated for display on VR HMDs, have been shown to enhance the surgeon’s understanding of complex pulmonary anatomy and facilitate precise surgical interventions.

Recent advances in 3D reconstruction from digitalized images have enabled the provision of intraoperative surgical navigation, as evidenced in our pilot study [2,3,4,5]. We found that the anatomical accuracy of these models significantly improves the safety and reliability of operative procedures. The VR HMD setup, in comparison to conventional preoperative imaging review, offers several advantages, including the ability for surgeons to interact more intuitively with the 3D reconstructions, thanks to the 3D vision already present in the robotic viewer. 

In our study, we identified several key advantages of using the VR HMD setup compared to traditional preoperative imaging methods. Utilizing the Perspectus VR Education software platform, we were able to generate 3D VR images that significantly enhanced the visualization of pulmonary anatomy before surgery. This approach allowed surgeons to interact more intuitively with the reconstructions, leveraging the 3D capabilities already inherent in the robotic viewer. Our findings suggest that an ideal pulmonary resection plan derived from preoperative 3D VR simulations can be seamlessly integrated into the robotic surgical approach. This integration of advanced visualization techniques significantly enhances the surgeon’s ability to plan for the procedure. It enables surgeons to view the lung model from any desired orientation relative to the thoracoscope and surgical instruments. Particularly, the ability to easily identify anomalous pulmonary vessels allows for more effective planning to manage these structures, enhancing the safety and precision of the surgery.

A notable innovation of our study is the software-based integration of the virtual model within the surgical console during RATS. This integration, which allows the virtual image to overlap with the endoscopic view, accurately identified tumor locations, thereby enhancing the surgeon’s ability to make informed decisions during the procedure. This multimodal imaging system protocol effectively combines preoperative anatomical assessments with intraoperative needs, placing critical information directly at the surgeon’s disposal within the surgical console.

These developments in computer science and their integration into medical practice open new avenues for preoperative planning, particularly for complex surgical procedures. The ability to smoothly execute technically challenging operations and improve patient safety represents a significant advancement in the field. This integration of advanced VR technology into the surgical workflow not only enhances the technical performance of complex procedures but also has the potential to significantly improve patient outcomes. Overall, our study underscores the growing importance and utility of VR technology in modern thoracic surgery, paving the way for more precise, safe, and effective surgical interventions.

As demonstrated in our previously published work [4], the visualization of 3D models significantly enhances a surgeon’s understanding of the disease, offering a clearer view of the patient’s anatomy and pathology. This advancement, we believe, marks a new paradigm in the era of precision robotic surgery [9]. In our recent study, we primarily used the Perspectus VR Education software as a preoperative simulation tool. However, its potential extends beyond this to include real-time intraoperative navigation, especially when integrated with the TilePro™ platform.

Theoretically, if automated registration of surgical console images to the 3D reconstructions were incorporated into the Perspectus VR Education software, surgeons could perform real-time surgical resections with dynamic overlays provided by the software. This advanced level of integration, which allows manipulation of the 3D models on a laptop, is uniquely feasible in the context of robotic surgery. The surgical console, designed to immerse the surgeon in the local conditions of the operation, plays a crucial role in reducing informational overload. It allows surgeons to naturally project and understand intricate anatomy without additional mental effort, thereby significantly enhancing the decision-making process during critical phases of the surgery, such as during dissections and the application of staples to various anatomical structures.

We envision a comprehensive surgical approach that combines a multimodal imaging system with a robotic platform, potentially preventing intraoperative accidents and reducing postoperative complications while also improving the overall performance of surgical procedures. Looking ahead, it is likely that these technologies will be integrated directly into robotic systems, enabling real-time 3D reconstruction and further revolutionizing the field of robotic surgery. This integration promises to enhance the safety and accuracy of thoracic surgery, especially in complex cases and represents a significant step forward in the evolution of surgical technology.

In the realm of simulation training, the use of VR 3D reconstruction, as facilitated by the Perspectus VR Education software, offers substantial benefits. Preoperative review of a patient’s individual anatomy, tumor size, and location through VR simulation is invaluable for assessing surgical respectability during multidisciplinary case discussions. Additionally, VR 3D reconstruction serves as a crucial educational tool for trainees, allowing them to navigate and review 3D models without the need for the same level of interpolation required by standard cross-sectional imaging. This approach enables trainees to familiarize themselves with basic anatomy and better prepare for complex surgical cases.

The multi-user functionality of the Perspectus VR Education software further enhances its educational value. It allows both trainees and senior surgeons to cooperatively operate the same model within a shared space, facilitating interactive discussions and collaborative learning akin to dual console surgery. Simulation-based training, particularly with VR, is known to reduce the learning curve by enabling surgical trainees to practice technical skills in a representative and time-efficient manner, thereby accelerating the acquisition of surgical capabilities [10]. This type of training prepares trainees for a variety of procedures and potential intraoperative complications, with evidence suggesting that training with VR simulators can improve performance in the operating room [10]. While several VR simulators have been used in robotic surgeries, their impact on training, especially in terms of skill transfer, is still being explored. Among the commercially available VR simulators, only a few offer procedure-based training, such as the dV-Trainer, RobotiX Mentor, RoSS, and SEP Robot, with initial validation supporting their content [11,12,13,14]. However, these simulators are often expensive and challenging to set up in each hospital. In contrast, our simulation system utilizing the Perspectus VR Education software offers a more cost-effective and accessible alternative, as it requires only commercially available equipment and software. This accessibility could significantly broaden the scope and reach of VR-based surgical training, potentially transforming the way surgical skills are acquired and homed in the field of robotic surgery.

An ideal multimodal imaging system in thoracic surgery should seamlessly provide critical anatomical information exactly where and when it is needed in the operating room. Achieving this level of integration, particularly in RATS, is feasible primarily through the surgical console. Nonetheless, it’s crucial to balance the wealth of information available to avoid overwhelming the operator. The information presented must be both relevant and timely to ensure it aids rather than hinders the surgical process.

Other advanced technology has been used in various surgical fields. Three papers collectively examined the application of extended reality (XR), augmented reality (AR), and VR technologies with a focus on enhancing surgical precision and patient outcomes. The first paper showcases the effectiveness of XR in laparoscopic liver resections [15]. XR technology has improved surgical visualization and anatomical recognition, leading to successful outcomes without the need to convert to open surgery. This study underscores the benefits of XR in enhancing surgical precision and patient safety. A significant aspect of this study is its innovative use of XR in a clinical setting, demonstrating its potential to revolutionize surgical procedures by improving visualization and decision-making during surgery. The second paper discusses the role of AR in neurosurgery, particularly in the management of brain tumors [16]. It highlights advancements in imaging and surgical tools that have propelled progress in brain tumor treatment and emphasizes the potential of AR to improve surgical precision and outcomes further. AR shows promise for guiding neurosurgical procedures, pinpointing tumor locations, and minimizing risks during surgery. The third paper explores the applications of VR and AR in orthopedic trauma surgery, from diagnosis to rehabilitation, highlighting the transformative potential and current challenges of these technologies in improving medical procedures [17]. It discusses how VR and AR can aid in pre-surgery planning, enhance surgical precision during operations, and assist in post-surgery rehabilitation. The paper provides a comprehensive overview of the current state and future potential of VR/AR technologies in orthopedic trauma surgery, offering insights into how these technologies could revolutionize patient care and treatment outcomes. We believe that these studies will be applicable and beneficial in the future for thoracic surgery, especially in the realm of robotic surgery.

We believe that the integration of a virtual reality (VR) head-mounted display (HMD) system with a robotic surgical platform represents a significant advancement in surgical methodology. This combined approach has the potential to substantially mitigate both intraoperative and postoperative complications. The ability to characterize a patient’s individual pulmonary anatomy with high precision is particularly beneficial in complex thoracic surgeries, such as intricate segmentectomies. This precise visualization not only aids in the surgical procedure itself but also contributes greatly to the safety and accuracy of the operation. By providing surgeons with detailed, patient-specific anatomical information in an intuitive and manageable format, such a system represents a significant advancement in the field of thoracic surgery, paving the way for more precise and safer surgical interventions in complex cases.

## 5. Conclusions

In conclusion, our initial experience with the Perspectus VR Education software and VR simulation using HMDs demonstrates its feasibility and effectiveness in preoperative planning for RATS. By integrating routine preoperative imaging with VR technology, we have found that it is possible to significantly enhance the planning process, thereby improving both the safety and accuracy of anatomical resections.

Our findings indicate that virtual reconstructions created with the Perspectus VR Education software enable surgeons to perform complex RATS procedures, such as segmentectomies, effectively. This technology not only aids in the surgical process itself but also shows great promise in revolutionizing the training for robotic surgery. The immersive and interactive nature of VR with HMD provides a more intuitive and comprehensive understanding of complex anatomical structures, which is crucial for both novice and experienced surgeons.

Looking forward, the integration of VR technology in thoracic surgery holds immense potential. It stands to not only enhance the capabilities of surgeons in performing intricate procedures but also to transform the way surgical training is conducted, paving the way for more advanced, precise, and safer surgical practices in the field of thoracic surgery.

## Data Availability

The data underlying this article will be shared on reasonable request to the corresponding author.

## Supplementary Materials

The following supporting information can be downloaded at: https://www.mdpi.com/article/10.3390/jcm13020611/s1, Video S1: The preoperative review process is significantly enhanced by the advanced functionalities offered by the Perspectus VR Education software. This software platform is specifically designed to cater to the intricate needs of surgical planning. One of its key features is the ability to adjust density and intensity thresholds in real time. This functionality allows surgeons to tailor the 3D model to their specific requirements, ensuring that all relevant anatomical details are highlighted and easily interpretable.; Video S2: Intraoperative viewing of the 3D VR models was performed at the surgeon’s discretion.; Video S3: The left lingular segmentectomy described here was conducted to resect a nodular lesion located in the left lung’s segment 4 + 5, measuring 15 mm in diameter. A key aspect of this procedure was the utilization of a virtual reality surgical navigation system equipped with head-mounted displays (VR HMD). We employed indocyanine green (ICG) for firefly fluorescence imaging during the surgery. This technique was instrumental in clearly demarcating the intersegmental border. The use of ICG fluorescence imaging enhances the visibility of the lung segments, allowing for precise identification and preservation of the lung tissue.
